# Genotype-Related Clinical Characteristics and Myocardial Fibrosis and Their Association with Prognosis in Hypertrophic Cardiomyopathy

**DOI:** 10.3390/jcm9061671

**Published:** 2020-06-01

**Authors:** Hyung Yoon Kim, Jong Eun Park, Sang-Chol Lee, Eun-Seok Jeon, Young Keun On, Sung Mok Kim, Yeon Hyeon Choe, Chang-Seok Ki, Jong-Won Kim, Kye Hun Kim

**Affiliations:** 1 Department of Cardiovascular Medicine, Chonnam National University Medical School/Hospital, Gwangju 61469, Korea; medoc7@gmail.com (H.Y.K.); christiankyehun@hanmail.net (K.H.K.); 2 Department of Laboratory Medicine, Hanyang University Guri Hospital, Hanyang University College of Medicine, Guri 11923, Korea; jongeun820@gmail.com; 3 Department of Internal Medicine, Cardiovascular Imaging Center, Heart, Vascular & Stroke Institute, Samsung Medical Center, Sungkyunkwan University School of Medicine, Seoul 06351, Korea; eunseok.jeon@samsung.com (E.-S.J.); yk.on@samsung.com (Y.K.O.); 4 Department of Radiology, Cardiovascular Imaging Center, Heart, Vascular & Stroke Institute, Samsung Medical Center, Sungkyunkwan University School of Medicine, Seoul 06351, Korea; sungmok_kim@hanmail.net (S.M.K.); ychoe11@gmail.com (Y.H.C.); 5 Green Cross Genome, Yongin 16924, Korea; changski.md@gmail.com; 6 Department of Laboratory Medicine and Genetics, Samsung Medical Center, Sungkyunkwan University School of Medicine, Seoul 06351, Korea; kimjw@skku.edu

**Keywords:** hypertrophic cardiomyopathy, genotype, phenotype, sarcomeric

## Abstract

Background: The spectrum of genetic variants and their clinical significance of Hypertrophic cardiomyopathy (HCM) have been poorly studied in Asian patients. The objectives of this study were to assess the spectrum of genetic variants and genotype–phenotype relationships within a Korean HCM population. Methods: Eighty-nine consecutive unrelated HCM patients were included. All patients underwent genotypic analysis for 23 HCM-associated genes. Clinical parameters including echocardiographic and cardiac magnetic resonance (CMR) parameters were evaluated. A composite of major adverse cardiac and cerebrovascular events was assessed. Results: Genetic variants were detected in 55 of 89 subjects. Pathogenic variants or likely pathogenic variants were identified in 27 of HCM patients in *MYBPC3*, *TNNI3*, *MYH7*, and *MYL7*. Variants of uncertain significance were identified in 28 patients. There were significant differences in the presence of non-sustained ventricular tachycardia (*p =* 0.030) and myocardial fibrosis on CMR (*p =* 0.029) in the detected compared to the not-detected groups. Event-free survival was superior in the not-detected group (*p =* 0.006). Conclusion: Genetic variants in patients with HCM are relatively common and are associated with adverse clinical events and myocardial fibrosis on CMR. Genotypic analysis may add important information to clinical variables in the assessment of long-term risk for HCM patients.

## 1. Introduction

Hypertrophic cardiomyopathy (HCM) is a genetic heart disease with an estimated prevalence of 0.2% of the global population [1]. The clinical manifestations of HCM are highly variable and range from asymptomatic left ventricular (LV) hypertrophy to progressive heart failure and sudden cardiac death (SCD).

Identification of disease-causing variants and their particular phenotypic expression and natural history has been attempted in many molecular and clinical genetic studies yet much remains unclear. To date, more than 1500 different variants in at least 14 genes have been reported in patents with HCM [2,3]. However, the association between the genetic variants and phenotypic expression in HCM remains controversial [4,5,6]. Recent guidelines advise the use of genotypic analysis in HCM patients in clinical practice, but the prognostic significance of genetic testing is still under debate [7,8]. With advances in techniques for genotypic analysis and tools for clinical evaluation such as cardiac magnetic resonance (CMR) imaging, which provides not only accurate information on LV morphology but also information on the amount and pattern of myocardial fibrosis in HCM [9,10], novel approaches for the evaluation of genotypic–phenotypic associations and risk evaluation in HCM are required.

The aims of this study were to understand the distribution of genetic variants among Korean HCM patients and to link their clinical and imaging phenotypic characteristics by genotype status.

## 2. Materials and Methods

### 2.1. Subjects and Design of the Study

This study is based on subjects with HCM from the prospective HCM registry of the Samsung Heart Vascular Stroke Institute, Seoul, Korea. Patients with diagnosis of HCM based on echocardiography were consecutively enrolled. Inclusion criteria were (i) end-diastolic LV wall thickness of ≥15 mm in any site or LV septal thickness:posterior wall thickness ≥ 1.3 with the absence of any underlying condition that may be associated with LV hypertrophy (i.e., long-standing uncontrolled systemic hypertension, aortic or subaortic stenosis, and metabolic storage disease or inflammatory disease); (ii) end-diastolic LV septal thickness:posterior wall thickness ≥1.5 in patients with systemic hypertension; or (iii) LV hypertrophy confined to the LV apex (apical 4 segments and apical cap according to the 17-segment guidelines of the American Society of Echocardiography) with maximal apical wall thickness of ≥15 mm or a ratio of maximal apical to posterior wall thickness of ≥1.3 at end-diastole, regardless of the presence of systemic hypertension [7,11,12,13].

We excluded subjects with poorly controlled hypertension, uncontrolled ventricular arrhythmias, severe valvular diseases, and other concomitant systemic diseases including malignancy and those who were contraindicated for cardiovascular magnetic resonance imaging (CMR) or had poor echocardiographic windows for analysis. We also excluded those who were diagnosed on echocardiography as having non-compaction cardiomyopathy, defined as the presence of prominent trabeculation with an increased ratio of non-compacted to compacted LV segments.

All patients provided written informed consent, and Institutional Review Board approval was obtained by the institutional review board of Samsung Medical Center, Sungkyunkwan University School of Medicine (IRB No. 2015-06-076-001).

### 2.2. Genetic Testing and Data Analysis

Genomic DNA was extracted from peripheral blood leukocytes of each patient using MagNA Pure 96 instrument (Product No. 6640729001, Roche Diagnostics, Mannheim, Germany) with MagNA Pure 96 DNA and Viral NA Large Volume Kit (Product No. 6374891001, Roche Diagnostics, Mannheim, Germany) following the manufacturer’s instructions. Of the 89 total patients, samples from 77 were enriched using the TruSeq Custom Enrichment Kit and sequenced with a MiSeq (Illumina, Inc., San Diego, CA, USA), and the other 12 were enriched using the AmpliSeq custom panel (Thermo Fisher Scientific) and sequenced with an Ion Torrent S5 XL (Thermo Fisher Scientific, Waltham, MA, USA). Sequence reads were aligned to the Human Reference Genome (NCBI build GRCh37) using Burrows-Wheeler Aligner (BWA) 0.7.12; duplicated reads were marked using by Picard Tools v1.130; local-realignment, base quality recalibration, and variant calling were performed using the Genome Analysis ToolKit (GATK, Broad Institute, MA, USA) v3.4.0; and variant annotation and effect predictions were performed using SnpEff v4.1g.

The number of candidate variants was filtered and prioritized using a four-step strategy. Initially, we included variants over 10× depth of coverage. Based on HCM prevalence, the minor allele frequency (MAF) below 5% in the Genome Aggregation Database (gnomAD) or Korean Reference Genome Database (KRGDB) were included [14,15]. The third step was to prioritize variants which affect protein coding region such as missense, nonsense, frameshift, or in-frame insertion/deletion variants or consensus splice site variants. Finally, gene-specific analysis was performed with an in silico gene panel consisting of 23 genes for HCM. These genes have been described in previous reports [5,16,17] (Table 1).

### 2.3. Criteria for Evidence-Based Classification of Candidate Variants

Candidate variants were interpreted using the standards and guidelines of the American College of Medical Genetics and Genomics (ACMG) and the Association for Molecular Pathology (AMP) [18]. These guidelines list five categories of variants: pathogenic variant (PV), likely pathogenic variant (LPV), variant of uncertain significance (VUS), likely benign variant (LBV), and benign variant (BV). These variants were categorized based on a combination of multiple weighted factors, such as the population database, as well as functional and computational data. We used gnomAD and KRGDB to check the frequency of each variant in the general population. Potential pathogenicity was assessed using the Human Gene Mutation Database (HGMD) professional version, release January 2018 and ClinVar, SIFT and Polyphen2 were used to predict the impact of missense changes [19,20,21,22].

### 2.4. Data Collection

Baseline demographic and clinical data included age, sex, cardiovascular risk factors such as presence of hypertension, diabetes mellitus or smoking history, and clinical presentations. Patients were evaluated for first-degree familial history of HCM or SCD. Dyspnea was categorized with New York Heart Association (NYHA) functional classification. Additionally, 24-h Electrocardiography (ECG) monitoring and a symptom-limited treadmill test were performed to identify the risk of SCD. Abnormal exercise blood pressure (BP) responses were defined as follows: (i) increase in systolic BP (sBP) < 20 mmHg during or after exercise; (ii) decrease in sBP < 20 mmHg during or after exercise, without an initial increase in sBP; (iii) decrease in sBP ≥ 20 mmHg during exercise after initial rise; or (iv) an initial increase in sBP with a subsequent fall of sBP > 20 mm Hg compared with peak sBP [23,24,25,26].

### 2.5. 2D Echocardiography 

Standard two dimensional (2D) echocardiographic images were acquired with standard methods using a commercially available system (Vivid 7, GE Medical System, Horten, Norway). LV chamber size and wall thickness were measured by M-mode echocardiography using the 2005 American Society of Echocardiography (ASE) guideline and standards [27]. LV ejection fraction was estimated by modified Simpson’s method from apical imaging planes. LV mass was calculated using the conventional cube formula. LV outflow tract (LVOT) and mid-ventricular (MV) obstruction were defined as peak pressure gradient >30 mmHg [11].

**Figure 1:**
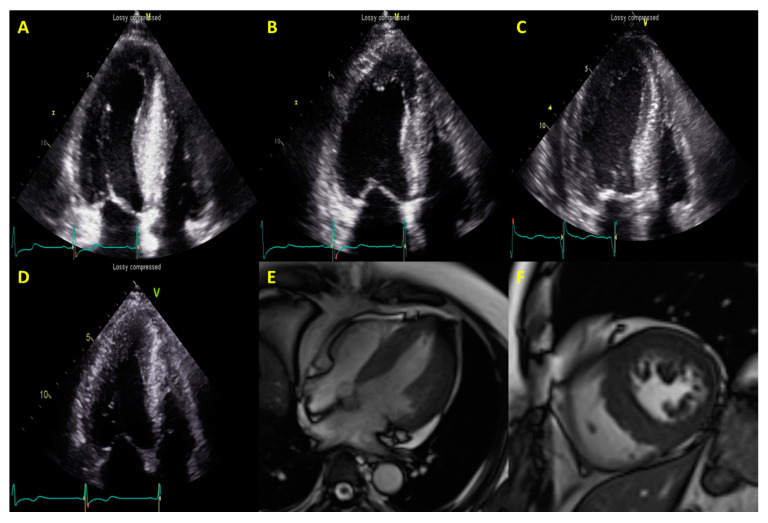
Sample illustration of imaging phenotypes of Hypertrophic cardiomyopathy (HCM) on echocardiography: septal hypertrophy (**A**), septal and apical hypertrophy (**B**), concentric hypertrophy (**C**), apical hypertrophy (**D**), and representative cardiac magnetic resonance (MR) images of HCM (**E**,**F**).

HCM were classified into four types according to the pattern and degree of LV hypertrophy under the following criteria: (i) septal hypertrophy alone; (ii) septal and apical hypertrophy; (iii) concentric hypertrophy involving the entire LV myocardium; and (iv) apical hypertrophy alone (Figure 1).

### 2.6. Acquisition and Analysis of CMR Images

All study patients underwent CMR using an identical protocol on a 1.5T MR scanner (MAGNETOM Avanto; Siemens Medical Solutions, Erlangen, Germany) (Figure 1). After localization, cine images of the left ventricle (LV) were acquired using a steady-state free precession (SSFP) sequence on 4-, 3-, and 2-chambers and short-axis (SA) views for obtaining 8–10 contiguous SA slices to include the entire LV with 6-mm slice thicknesses and 4-mm intersection gaps. Late gadolinium enhancement (LGE) on CMR was performed 10 min after injection of 0.15 mmol/kg bodyweight of Gadobutrol (Gadovist^®^; Bayer Healthcare, Berlin, Germany) using a multi-shot, turbo field echo, breath-hold sequence with a phase-sensitive inversion recovery method. The inversion time (TI) was adjusted using a Look-Locker-based TI scout sequence to determine the proper TI to null normal myocardium.

All imaging analysis was performed at the Samsung Medical Center CMR core laboratory with a dedicated workstation (CAAS MRV version 1.0, Pie Medical Imaging B.V., Maastricht, The Netherlands). The images were analyzed by two experienced CMR imaging specialists who were blinded to other data, and discrepancies in interpretation were reconciled during consensus reading. Endocardial and epicardial borders were traced manually on short-axis images at end-diastolic and end-systolic phases, respectively. Papillary muscles and LV trabeculae were excluded from the endocardium and included in the LV cavity volume. For quantification of fibrosis, LGE was defined as areas with a signal intensity > 6 standard deviations (SD) above the mean signal intensity of remote normal myocardium.

LGE volume was calculated by summing areas with hyper-enhancement of all short-axis slices and expressed as a volumetric proportion of LGE to the total LV myocardium (% LV).

### 2.7. Outcomes

Clinical outcomes were investigated by medical record review. Major adverse cardiac and cerebrovascular events (MACCE) were defined as all-cause death, sudden cardiac death, sustained ventricular tachycardia/ventricular fibrillation (VT/VF) with or without implantable cardioverter defibrillator (ICD) implantation, hospitalization due to worsening of heart failure (HF) at NYHA class III or IV, hospitalization due to new onset atrial fibrillation (AF), stroke, syncope, and acute myocardial infarction (AMI).

### 2.8. Statistical Analysis

Categorical variables are presented as frequencies and percentages. The Chi-square test or Fisher’s exact test was performed appropriately to compare categorical variables. Continuous variables are presented as mean ± SD to analyze the difference between numeric variables. The Student’s *t*-test or Mann–Whitney test was performed to test for differences in continuous variables. All tests were 2-tailed, and a *p* value of less than 0.05 was considered significant. Pearson’s and Spearman’s correlation coefficients were calculated to determine the relationship between two variables. The probability of freedom-from-MACCE was estimated according to the Kaplan–Meier method and compared using the log-rank test. Data were analyzed using SPSS statistical software (version 20.0 for windows, SPSS, Inc., Chicago, IL, USA).

Patients were categorized into two groups according to identification of their involved variants: the detected group and the not-detected group. “Detected” was defined as the presence of at least one variant classified as PV, LPV, or VUS.

## 3. Results

### 3.1. Study Population and Baseline Clinical Characteristics 

Between March 2013 and February 2017, a total of 100 unrelated consecutive subjects with HCM were included in this study. Among them, eight patients with absent or poor CMR image quality and three patients with non-compaction were excluded. A total of 89 patients were finally analyzed. Their mean age at diagnosis was 48.5 ± 10.5 years, and 85.4% (*n* = 76) were male. Median follow-up duration was 47.3 months (range 2 to 106.3 months). Demographic and clinical characteristics of the study population are summarized in Table 2, and prescribed medications are summarized in Table S1.

### 3.2. Variant Profile of HCM Patients

**Figure 2:**
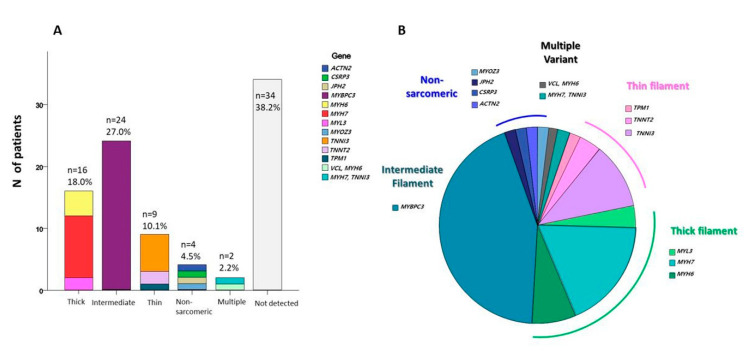
Distribution of affected variants identified for each analyzed gene: (**A**) Distribution of affected variants classified by gene variant location and (**B**) subgroup analysis of distribution of affected variants among the detected variants.

**Figure 3:**
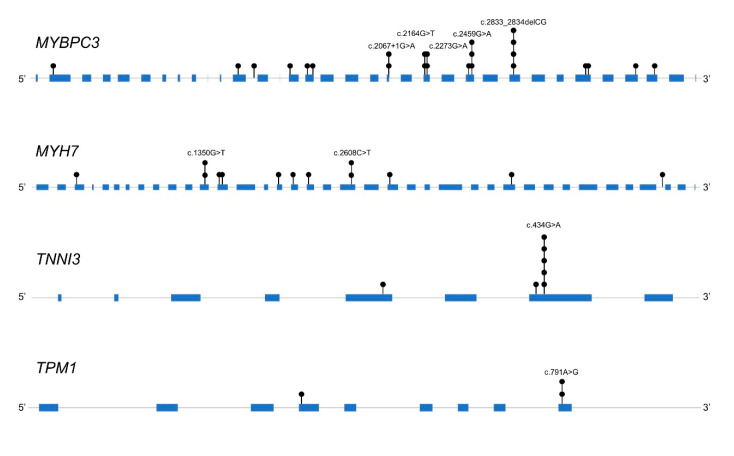
Distribution of variants in a gene where overlapping variants were detected.

Among the 89 HCM patients, 55 (61.7%) were found to carry at least one rare variant. PV or LPV were identified in 27 (30.3%) patients, and 28 VUSs were identified in 28 (31.5%) patients. Within the detected group, most of the changes mapped to sarcomeric loci (92.7%, *n* = 51), including alterations of *MYBPC3* (MIM: 160781) (43.6%), *MYH7* (MIM: 160760) (18.2%), *TNNI3* (MIM: 191044) (10.9%), *MYH6* (MIM: 160710) (7.3%), and *TNNT2* (MIM: 191045) (3.6%), in order of decreasing frequency. Thick filament genes were found to be 18.0%, intermediate filament genes were 27.0%, thin filament genes were 10.1%, and non-sarcomeric genes were 4.5%. The distribution of identified variants for each analyzed gene is shown in Figure 2 and Table 3. Among the detected variants, overlapping ones were identified *in MYBPC3, MYH7, TNNI3*, and *TPM1* genes (Figure 3).

### 3.3. Differences in Baseline Clinical Characteristics between HCM Patients in Detected vs. Not-Detected Groups

Mean age and sex ratio were not significantly different between the detected and not-detected groups. The presence of CV risk factors such as presence of hypertension, diabetes mellitus, and history of smoking also did not differ. There were no statistical differences in patient history of syncope or familial history of HCM or SCD. NYHA functional class status was similar between the two groups, which corresponded with similar mean levels of plasma N-terminal prohormone of brain natriuretic peptide (NT-proBNP).

In the detected group, palpitation was more frequent, (*p* = 0.040) and non-sustained ventricular tachycardia (NSVT) was more commonly present on 24-h ECG monitoring (24.5% vs. 5.9%; *p* = 0.030).

Abnormal hemodynamic response to exercise was observed in 39.3% of patients, with no statistical difference between the groups. Other ECG findings such as the presence of T wave inversion, atrial fibrillation, or the distribution of the pattern of HCM were also similar.

### 3.4. Imaging Phenotypes of HCM Patients in Detected vs. Not Detected Groups

The average maximal LV wall thickness was 17.14 ± 4.0 mm, and mean LV mass index (LVMI) was 123.0 ± 37.8 (g/m^2^). LV geometry such as LV wall hypertrophy, LV volumes, LV mass, and LV ejection fraction as assessed by both echocardiography and CMR were similar between the two groups. Echocardiographic and CMR findings are summarized in Table 4.

Septal hypertrophy (40.4%) was the most common morphological subtype, followed by septal and apical (34.8%), apical (16.9%), and concentric (5.6%) subtypes, and no statistical difference was detected in the distribution of morphological subtypes between the groups. 

LV cavity obstruction was found in 27 patients (30.3%), with LVOT obstruction in 16 patients, MV obstruction in 13 patients, and systolic anterior motion of mitral valve (SAM) in 16 patients (18.0%). In particular, LVOT obstruction and SAM were most commonly found in the septal type of HCM (75.0%, *p* = 0.008, and 62.5%, *p* = 0.083, respectively). However, no differences were demonstrated in the presence of LV cavity obstruction or SAM between the detected and not-detected groups.

LGE on CMR was detected in 86.5% of patients with HCM, and the amount of LGE showed significant correlation with maximal wall thickness (*p* < 0.001) and LVMI (*p* < 0.001) on 2D echocardiography.

LGE was present more frequently in the detected group than in the not-detected group (92.7% vs. 76.5%; *p* = 0.029). Accordingly, the volume percent of LGE was greater in the detected group (9.12% ± 6.7% vs. 6.06% ± 4.9%; *p* = 0.021).

### 3.5. Clinical Outcomes

During the follow-up period, 25 patients experienced a composite MACCE, including 4 cases of sustained VT/VF, 7 cases of new onset AF, 7 cases of HF aggravation, 2 cases of stroke, 4 cases of syncope, and 1 case of AMI. No mortality occurred during the follow-up period. 

The incidence of composite events was higher in the detected group than in the not-detected group (38.2% vs. 11.8%, *p* = 0.008). Interventions such as ICD implantation, septal myectomy, and heart transplantation were more frequently performed in patients in the detected group, but the difference was not statistically significant (16.4% vs. 2.9%, *p* = 0.082). Distribution of MACCE and interventions are summarized in Table 5.

**Figure 4:**
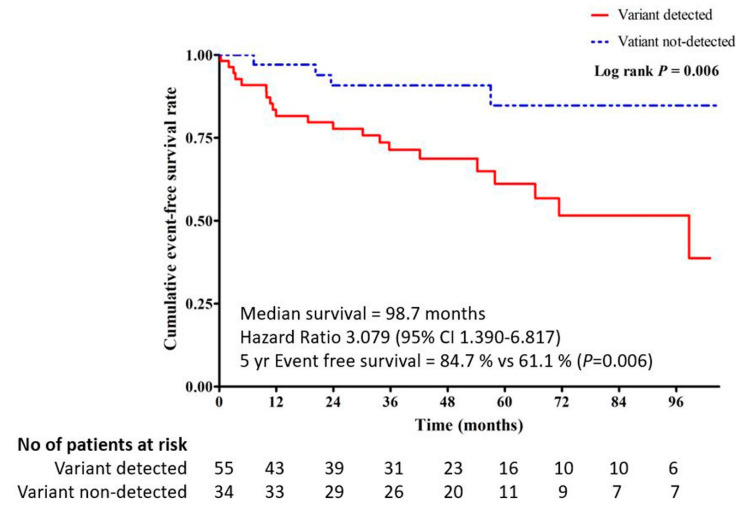
Cumulative event-free survival curves stratified by the variant detected or not-detected groups: Event-free survival was significantly different between the two groups (*p* = 0.006). CI: confidence interval.

Overall event-free survivals were 94.4%, 88.6%, 78.1%, and 67.7% at 1, 3, 5, and 10 years, respectively. Notably, the not-detected group had better event-free survival than the detected group (5-year survival rate 84.7% vs. 61.1%, *p* = 0.012). Kaplan–Meier event-free survival curves for MACCE are shown in Figure 4.

## 4. Discussion

In this single-center prospective registry, we investigated the distribution of genetic variants in Korean HCM patients and associated differences in clinical and imaging phenotypic characteristics. Patients with significant variants showed higher frequencies of poor prognostic factors such as NSVT on 24-h ECG and LGE in CMR as well as worse prognosis during a mid-range follow-up period. 

Since the first genetic variant in HCM was documented, many additional variants have been identified over the last 30 years [28,29,30]. In the current study of Korean HCM patients, we found that more than 60% of patients had identified variants when a comprehensive gene panel (23 genes) was used. The majority of variants were located at the sarcomeric loci, which accounted for 92.7% of variant detected cases, and most were present in *MYBPC3* (43.6%) and *MYH7* (19.2%), followed by other variants of *TNNI3* (10.9%), *MYH6* (7.3%), and *MYL3* (MIM: 160790) (3.6%). This distribution of variants according to gene location was similar to previous studies on sarcomeric variants, in which the prevalence of variants in *MYBPC3* and *MYH7* account for 75–80% of the genetic basis of HCM [3,6].

Non-sarcomeric variants might be causal variants, but data confirming pathogenicity are lacking. There are few studies concerning non-sarcomeric variants, and reported locations include *ACTN2* (MIM: 102573), *ANKRD1* (MIM: 609599), *CALR3* (MIM: 611414), *CAV3* (MIM: 601253), *CRYAB* (MIM: 123590), *CSRP3* (MIM: 600824), *DES* (MIM: 125660), *JPH2* (MIM: 605267), *LDB3* (MIM: 605906), *MYLK2* (MIM: 606566), *MYOZ2* (MIM: 605602), *MYPN* (MIM: 608517), *NEXN* (MIM: 613121), *PLN* (MIM: 172405), *TCAP* (MIM: 604488), and *VCL* (MIM: 193065) [31,32]. In our gene panel, 13 out of the 23 genes consisted of non-sarcomeric *loci*: *ACTN2*, *ANKRD1*, *CAV3*, *CRYAB*, *CSRP3*, *JPH2*, *LDB3*, *MYLK2*, *MYOZ2*, *NEXN*, *PLN*, *TCAP*, and *VCL*. Non-sarcomeric variants accounted for only 7.2% of the total detected variants, making it difficult to interpret their influence on disease phenotype. The low prevalence of non-sarcomeric variants in the current study is similar to a recent meta-analysis by Walsh et al. [31].

Current risk estimation for sudden cardiac death for HCM is based on clinical features, including age, familial history of SCD, maximal wall thickness, left atrial diameter, maximal LVOT gradient, NSVT on 24-h ECG, and unexplained syncope [33]. In the present study, we investigated most of the alleged risk factors, and the frequency of NSVT was significantly higher in patients with detected variants. This finding is in accord with a previous study by Farbod et al., in which the frequency of VT was significantly higher in patients with an *MYH7* variant compared to the variant negative group [34].

In our study, LV myocardial fibrosis on CMR was significantly more severe and prevalent in patients with detected variants. CMR has been reported to be superior to standard echocardiography in the detection of subtle evidence of disease and early information of myocardial deformation in patients with sarcomeric variants [35]. Meanwhile, LV morphological and functional status assessed by echocardiography failed to demonstrate distinct characteristics according to genotypic status in this study. This result is consistent with previous reports, where LV geometry and LV systolic function were not associated with genotypic status [36,37]. The fact that the difference was detected only by CMR and not by echocardiography may be accounted for by the superior resolution and capability of tissue characterization in CMR, highlighting the need for newer studies on genotypic–phenotypic association.

In the current study, patients with detected variants were at increased risk of occurrence of MACCE, and event-free survival was higher in the not-detected group compared to the detected group, accordingly. There have been varying reports on the prognostic significance of genotypic analysis in HCM, with some studies showing poor prognosis in sarcomeric variant-positive patients, including increased risk for cardiac death and progression to NYHA class III or IV HF and stroke [29,38], while others show no prognostic significance of genetic variations [39,40]. In our study, as stated above, genetic variants showed associations with multiple alleged risk factors of HCM, especially the presence and amount of myocardial fibrosis. This may be related to the higher frequency of NSVT on 24-h ECG monitoring in the same group, which in previous studies has been reported to be associated with myocardial fibrosis as well [41,42]. These findings may partially explain the higher event rate in the detected group. As reports on the genotypic–phenotypic relationship in HCM based on CMR are scarce at this time but will likely be pursued more aggressively in the future [43,44], we predict that findings similar to ours will be found in larger studies in the near future.

It is also interesting to note that, among the events on follow-up, hospitalization associated with new-onset atrial fibrillation was especially more frequent in the detected group. As atrial fibrillation is gaining more attention as a problem of morbidity and an initiating cause of heart failure and stroke in HCM [45,46], more studies will be needed to address the association of genetic variants and atrial fibrillation.

There are several limitations in this study. First, a relatively small number of patients were included and the frequency of adverse events was low, resulting in limited statistical power. In addition, the properties of the non-sarcomeric variants that we included in this study may have affected the clinical phenotype of the detected group along with the sarcomeric variants, complicating the interpretation of how genetic status influences clinical prognosis. Control studies are needed for a more rigorous evaluation of the genotype–phenotype association in HCM. Lastly, there were only 23 genes included in the gene panel, which suggests that selection bias could exist in the not-detected population.

## 5. Conclusions

Nonetheless, our findings suggest that clinical presentation and prognosis are significantly worse in patients with HCM who possess pathogenic sarcomeric variants. As the risk stratification in HCM is highly problematic due to the genetic and clinical heterogeneity of the disease, incorporating both genotype and clinical phenotype analysis in assessment may be crucial in future studies of HCM and its consequences.

## Figures and Tables

**Table 1:** List of genes implicated in hypertrophic cardiomyopathy.

Gene	Protein	Location or Function	Chromosomal Position	MIM Number
*ACTC1*	Actin, alpha, cardiac muscle 1	Sarcomere	15q14	102540
*ACTN2*	Actinin alpha 2	Z-disk	1q43	102573
*ANKRD1*	Ankyrin repeat domain 1	Z-disk	10q23.31	609599
*CAV3*	Caveolin 3	Sarcolemma	3p25.3	601253
*CRYAB*	Crystallin alpha B	Z-disk	11q23.1	123590
*CSRP3*	Cysteine and glycine rich protein 3	Z-disk	11p15.1	600824
*JPH2*	Junctophilin 2	Intracellular calcium signaling	20q13.12	605267
*LDB3*	LIM domain binding 3	Z-disk	10q23.2	605906
*MYBPC3*	Myosin binding protein C, cardiac	Sarcomere	11p11.2	600958
*MYH6*	Myosin heavy chain 6	Sarcomere	14q11.2	160710
*MYH7*	Myosin heavy chain 7	Sarcomere	14q11.2	160760
*MYL2*	Myosin light chain 2	Sarcomere	12q24.11	160781
*MYL3*	Myosin light chain 3	Sarcomere	3p21.31	160790
*MYLK2*	Myosin light chain kinase 2	Phosphorylate myosin light chain 2	20q11.21	606566
*MYOZ2*	Myozenin 2	Z-disk	4q26	605602
*NEXN*	Nexilin F-actin binding protein	Z-disk	1p31.1	613121
*PLN*	Phospholamban	Regulator of sarcoplasmic reticulum calcium	6q22.31	172405
*TCAP*	Titin-cap	Z-disk	17q12	604488
*TNNC1*	Troponin C1, slow skeletal and cardiac type	Sarcomere	3p21.1	191040
*TNNI3*	Troponin I3, cardiac type	Sarcomere	19q13.4	191044
*TNNT2*	Troponin T2, cardiac type	Sarcomere	1q32.1	191045
*TPM1*	Tropomyosin 1	Sarcomere	15q22.2	191010
*VCL*	Vinculin	Z-disk	10q22.2	193065

**Table 2:** Baseline demographic and clinical characteristics.

Variables	Detected (*n* = 55)	Not Detected (*n* = 34)	*p*-Value
	Mean ± SD or Number (%)		
Age at diagnosis	48.69 ± 10.8	48.34 ± 10.3	0.880
Sex, male	45 (81.8)	31 (91.2)	0.225
Hypertension	12 (21.8)	11 (32.4)	0.270
Diabetes mellitus	5 (9.1)	1 (2.9)	0.261
Smoking			0.102
Current smoker	11 (20.0)	13 (38.2)	
Ex-smoker	15 (27.3)	10 (29.4)	
Familial history of HCM	9 (16.4)	5 (14.7)	0.835
Familial history of SCD	14 (25.5)	5 (14.7)	0.229
Chest discomfort/pain	13 (23.6)	15 (44.1)	0.060
Syncope	5 (9.1)	4 (11.8)	0.684
Palpitation	19 (34.5)	5 (14.7)	0.040 *
Dyspnea, NYHA class			0.457
I	43 (78.2)	24 (70.6)	
II	11 (20.0)	10 (29.4)	
III	1 (1.8)	0 (0.0)	
NT-proBNP (pg/mL)	568.9 ± 619.6	449.8 ± 493.2	0.188
ECG findings			
Atrial fibrillation	5 (9.1)	1 (2.9)	0.261
T-wave inversion	45 (81.8)	29 (85.3)	0.670
LBBB	0	0	
RBBB	2 (3.6)	2 (5.9)	0.619
LVH	27 (49.1)	21 (61.8)	0.244
PR interval	170.9 ± 24.7	161.7 ± 19.6	0.078
QRS duration	94.0 ± 11.5	102.1 ± 11.9	0.002 *
QTc interval	445.4 ± 31.2	458.4 ± 23.5	0.041 *
NSVT on Holter ECG	13 (24.5)	2 (5.9)	0.030 *
Abnormal BP response	23 (41.8)	12 (38.2)	0.546

Student’s *t*-test, Mann–Whitney test or χ2-test for detected vs. not detected (* *p*-value < 0.05). BP: blood pressure, ECG: electrocardiography, HCM: hypertrophic cardiomyopathy, LBBB: left bundle branch, LVH: left ventricular hypertrophy, NSVT: non-sustained ventricular tachycardia, NT-proBNP: N-terminal prohormone of brain natriuretic peptide, NYHA: New York Heart Association, RBBB: right bundle branch block, SCD: sudden cardiac death, SD: standard deviation.

**Table 3:** Pathogenic variants, likely pathogenic variants, and variants of uncertain significance in 23 sarcomeric and non-sarcomeric genes of hypertrophic cardiomyopathy patients.

Case No.	Gene	Refseq	Nucleotide Change	Protein Change	ACMG Interpretation ^a^	gnomAD ALL	gnomAD East Asian	KRGDB	Polyphen-2	SIFT
01-001-017	*MYBPC3*	NM_000256.3	c.1000G>A	p.(Glu334Lys)	LPV	2.54 × 10^−4^	3.55 × 10^−3^	4.00 × 10^−3^	Possibly damaging	Deleterious
01-001-018	*TNNT2*	NM_001001430.2	c.785A>G	p.(Asn262Ser)	VUS	4.76 × 10^−6^	0	N/A	Benign	Tolerated
01-001-026	*MYBPC3*	NM_000256.3	c.86delT	p.(Phe29Serfs*10)	LPV	N/A	N/A	N/A	N/A	N/A
01-001-041	*TNNI3*	NM_000363.4	c.433C>T	p.(Arg145Trp)	PV	4.07 × 10^−6^	0	N/A	Probably damaging	Deleterious
01-001-043	*MYBPC3*	NM_000256.3	c.2833_2834delCG	p.(Arg945Glyfs*105)	PV	4.10 × 10^−6^	5.86 × 10^−5^	N/A	N/A	N/A
01-001-046	*MYBPC3*	NM_000256.3	c.1483C>T	p.(Arg495Trp)	VUS	N/A	N/A	N/A	Possibly damaging	Deleterious
01-001-047	*MYBPC3*	NM_000256.3	c.2164G>T	p.(Glu722*)	LPV	N/A	N/A	N/A	N/A	N/A
01-001-049	*MYOZ2*	NM_016599.4	c.147T>A	p.(His49Gln)	VUS	N/A	N/A	N/A	Possibly damaging	Tolerated
01-001-061	*ACTN2*	NM_001103.3	c.440C>T	p.(Ser147Leu)	VUS	4.06 × 10^−6^	5.80 × 10^−5^	N/A	Probably damaging	Deleterious
01-001-062	*MYH7*	NM_000257.2	c.2069T>A	p.(Met690Lys)	VUS	N/A	N/A	N/A	Possibly damaging	Deleterious
01-001-064	*MYH7*	NM_000257.2	c.2608C>T	p.(Arg870Cys)	LPV	2.03 × 10^−5^	5.80 × 10^−5^	8.00 × 10^−4^	Possibly damaging	Deleterious
01-001-064	*TNNI3*	NM_000363.4	c.434G>A	p.(Arg145Gln)	LPV	1.63 × 10^−5^	1.16 × 10^−4^	N/A	Probably damaging	Tolerated
01-001-068	*MYH7*	NM_000257.2	c.2189T>C	p.(Ile730Thr)	VUS	N/A	N/A	N/A	Possibly damaging	Deleterious
01-001-069	*MYBPC3*	NM_000256.3	c.2833_2834delCG	p.(Arg945Glyfs*105)	PV	4.10 × 10^−6^	5.86 × 10^−5^	N/A	N/A	N/A
01-001-069	*MYH6*	NM_002471.3	c.5476_5477delinsAA	p.(Gly1826Asn)	VUS	N/A	N/A	1.60 × 10^−3^	Benign	Tolerated
01-001-071	*MYL3*	NM_000258.2	c.505G>C	p.(Val169Leu)	VUS	N/A	N/A	N/A	Benign	Deleterious
01-001-072	*MYH7*	NM_000257.2	c.400T>C	p.(Tyr134His)	VUS	N/A	N/A	N/A	Possibly damaging	Deleterious
01-001-074	*MYH7*	NM_000257.2	c.2608C>T	p.(Arg870Cys)	LPV	2.03 × 10^−5^	5.80 × 10^−5^	8.00 × 10^−4^	Possibly damaging	Deleterious
01-001-083	*MYBPC3*	NM_000256.3	c.2273G>A	p.(Gly758Asp)	VUS	3.23 × 10^−5^	6.17 × 10^−4^	N/A	Possibly damaging	Deleterious
01-001-085	*MYH6*	NM_002471.3	c.3413G>A	p.(Arg1138His)	VUS	4.31 × 10^−5^	6.10 × 10^−5^	N/A	Possibly damaging	Deleterious
01-001-086	*MYBPC3*	NM_000256.3	c.1358_1359delCT	p.(Pro453Argfs*2)	LPV	N/A	N/A	N/A	N/A	N/A
01-001-088	*MYBPC3*	NM_000256.3	c.2164G>T	p.(Glu722*)	LPV	N/A	N/A	N/A	N/A	N/A
01-001-090	*MYBPC3*	NM_000256.3	c.2441_2443delAGA	p.(Lys814del)	LPV	6.52 × 10^−5^	0	N/A	N/A	N/A
01-001-091	*TNNI3*	NM_000363.4	c.434G>A	p.(Arg145Gln)	LPV	1.63 × 10^−5^	1.16 × 10^−4^	N/A	Probably damaging	Tolerated
01-001-092	*MYBPC3*	NM_000256.3	c.3316G>A	p.(Asp1106Asn)	VUS	2.69 × 10^−5^	1.86 × 10^−4^	1.60 × 10^−3^	Possibly damaging	Deleterious
01-001-093	*MYBPC3*	NM_000256.3	c.2459G>A	p.(Arg820Gln)	PV	1.27 × 10^−5^	0	N/A	Possibly damaging	Deleterious
01-001-096	*MYBPC3*	NM_000256.3	c.3799delC	p.(Arg1267Alafs*64)	LPV	N/A	N/A	N/A	N/A	N/A
01-001-097	*MYH7*	NM_000257.2	c.1350G>T	p.(Lys450Asn)	VUS	N/A	N/A	N/A	Possibly damaging	Deleterious
01-001-099	*MYH7*	NM_000257.2	c.1988G>A	p.(Arg663His)	PV	8.12 × 10^−6^	0	N/A	Possibly damaging	Deleterious
01-001-102	*TPM1*	NM_001018005.1	c.376G>A	p.(Gly126Ser)	VUS	N/A	N/A	N/A	Possibly damaging	Tolerated
01-001-104	*CSRP3*	NM_003476.4	c.229G>A	p.(Ala77Thr)	VUS	2.03 × 10^−5^	1.74 × 10^−4^	N/A	Probably damaging	Deleterious
01-001-111	*MYBPC3*	NM_000256.3	c.1091-8G>A	p.(?)	VUS	8.49 × 10^−6^	1.18 × 10^−4^	8.00 × 10^−4^	N/A	N/A
01-001-115	*MYBPC3*	NM_000256.3	c.1624_1624+1delinsAGCTCAT	p.(Glu542_Gln1274delinsSerSer)	VUS	N/A	N/A	N/A	N/A	N/A
01-001-118	*JPH2*	NM_020433.4	c.1306C>T	p.(Arg436Cys)	VUS	1.64 × 10^−5^	2.34 × 10^−4^	2.40 × 10^−3^	Probably damaging	Deleterious
01-001-119	*MYBPC3*	NM_000256.3	c.2833_2834delCG	p.(Arg945Glyfs*105)	PV	4.10 × 10^−6^	5.86 × 10^−5^	N/A	N/A	N/A
01-001-125	*TNNI3*	NM_000363.4	c.434G>A	p.(Arg145Gln)	LPV	1.63 × 10^−5^	1.16 × 10^−4^	N/A	Probably damaging	Tolerated
01-001-126	*MYBPC3*	NM_000256.3	c.2067+1G>A	p.(?)	PV	N/A	N/A	1.60 × 10^−3^	N/A	N/A
01-001-128	*TNNI3*	NM_000363.4	c.434G>A	p.(Arg145Gln)	LPV	1.63 × 10^−5^	1.16 × 10^−4^	N/A	Probably damaging	Tolerated
01-001-129	*MYBPC3*	NM_000256.3	c.2459G>A	p.(Arg820Gln)	PV	1.63 × 10^−5^	5.80 × 10^−5^	N/A	Possibly damaging	Deleterious
01-001-130	*MYH6*	NM_002471.3	c.5026G>A	p.(Val1676Met)	VUS	2.03 × 10^−5^	5.80 × 10^−5^	N/A	Possibly damaging	Deleterious
01-001-131	*TNNT2*	NM_001001430.2	c.853G>A	p.(Gly285Arg)	VUS	2.93 × 10^−5^	5.91 × 10^−5^	N/A	Possibly damaging	Deleterious
01-001-137	*MYBPC3*	NM_000256.3	c.2459G>A	p.(Arg820Gln)	PV	1.63 × 10^−5^	5.80 × 10^−5^	N/A	Possibly damaging	Deleterious
01-001-139	*MYBPC3*	NM_000256.3	c.3626A>G	p.(Lys1209Arg)	VUS	N/A	N/A	N/A	Possibly damaging	Deleterious
01-001-141	*MYBPC3*	NM_000256.3	c.2273G>A	p.(Gly758Asp)	VUS	3.23 × 10^−5^	6.17 × 10^−4^	N/A	Possibly damaging	Deleterious
01-001-144	*MYH7*	NM_000257.2	c.1477A>G	p.(Met493Val)	VUS	N/A	N/A	N/A	Benign	Deleterious
01-001-145	*MYH7*	NM_000257.2	c.4130C>T	p.(Thr1377Met)	LPV	4.06 × 10^−6^	0	N/A	Probably damaging	Deleterious
01-001-148	*TNNI3*	NM_000363.4	c.434G>A	p.(Arg145Gln)	LPV	1.63 × 10^−5^	1.16 × 10^−4^	N/A	Probably damaging	Tolerated
01-001-155	*MYL3*	NM_000258.2	c.170C>G	p.(Ala57Gly)	LPV	7.31 × 10^−5^	2.90 × 10^−4^	1.60 × 10^−3^	Benign	Deleterious
01-001-159	*TPM1*	NM_001018005.1	c.791A>G	p.(Lys264Arg)	VUS	N/A	N/A	N/A	Benign	Tolerated
01-001-180	*MYBPC3*	NM_000256.3	c.2067+1G>A	p.(?)	PV	N/A	N/A	N/A	N/A	N/A
01-001-180	*MYH7*	NM_000257.2	c.2972A>G	p.(Lys991Arg)	VUS	4.06 × 10^−6^	0	N/A	Benign	Deleterious
01-001-181	*MYBPC3*	NM_000256.3	c.2833_2834delCG	p.(Arg945Glyfs*105)	PV	4.10 × 10^−6^	5.86 × 10^−5^	N/A	N/A	N/A
01-001-181	*MYH6*	NM_002471.3	c.2079C>A	p.(His693Gln)	VUS	4.06 × 10^−6^	0	N/A	Probably damaging	Deleterious
01-001-184	*MYH7*	NM_000257.2	c.1350G>T	p.(Lys450Asn)	VUS	N/A	N/A	N/A	Probably damaging	Deleterious
01-001-187	*TPM1*	NM_001018005.1	c.791A>G	p.(Lys264Arg)	VUS	N/A	N/A	N/A	Benign	Tolerated
01-001-190	*VCL*	NM_014000.2	c.3247G>A	p.(Glu1083Lys)	VUS	4.70 × 10^−6^	0	N/A	Probably damaging	Tolerated
01-001-190	*MYH6*	NM_002471.3	c.5071C>T	p.(Arg1691Cys)	VUS	1.22 × 10^−5^	1.74 × 10^−4^	5.80 × 10^−4^	Probably damaging	Deleterious
01-001-200	*MYH7*	NM_000257.2	c.1426C>T	p.(Leu476Phe)	VUS	N/A	N/A	N/A	Probably damaging	Deleterious
01-001-207	*MYBPC3*	NM_000256.3	c.3313_3314insGG	p.(Ala1105Glyfs*85)	LPV	N/A	N/A	N/A	N/A	N/A
01-001-223	*MYH7*	NM_000257.2	c.5560-8G>A	p.(?)	VUS	0.00 × 10^00^	0	N/A	N/A	N/A
01-001-228	*TNNI3*	NM_000363.4	c.235C>T	p.(Arg79Cys)	VUS	4.46 × 10^−4^	5.81 × 10^−3^	5.00 × 10^−3^	Probably damaging	Deleterious

gnomAD, Genome Aggregation Database; KRGDB, Korean Reference Genome Database; LPV, likely pathogenic variant; N/A, not applicable; PV, pathogenic variant; VUS, variant of uncertain significance; ^a^ Identified variants were classified according to the standards and guidelines of the American College of Medical Genetics and Genomics and the Association for Molecular Pathology [18].

**Table 4:** Distribution of echocardiographic and cardiac magnetic resonance imaging findings.

Variables	Detected (*n* = 55)	Not Detected (*n* = 34)	*p*-Value
	Mean ± SD or Number (%)		
Echocardiographic findings			
Maximal wall thickness (mm)	18.4 ± 4.3	17.5 ± 4.2	0.328
LVEDD (mm)	47.9 ± 5.4	49.0 ± 5.7	0.380
LVESD (mm)	26.7 ± 6.8	28.5 ± 3.5	0.102
IVSd (mm)	15.8 ± 4.9	14.7 ± 4.6	0.299
LVPWd (mm)	9.1 ± 2.3	9.6 ± 2.5	0.351
LV mass index (g/m^2^)	125.9 ± 31.9	118.2 ± 46.1	0.367
Relative wall thickness	0.385 ± 0.10	0.385 ± 0.12	0.604
LVEF (%)	66.67 ± 6.5	65.31 ± 6.76	0.353
LAVI (ml/m^2^)	42.5 ± 17.2	37.8 ± 10.3	0.119
E (m/sec)	0.62 ± 0.25	0.63 ± 0.23	0.822
A (m/sec)	0.62 ± 0.26	0.60 ± 0.17	0.717
Septal E’ (m/sec)	0.061 ± 0.022	0.057 ± 0.018	0.448
E/septal E’	15.6 ± 28.1	12.1 ± 4.31	0.659
RV involvement	9 (16.4)	5 (14.7)	0.543
LVOT obstruction	10 (18.2)	6 (17.6)	0.592
MV obstruction	8 (14.5)	5 (14.7)	0.606
LV gradient (mmHg)	22.1 ± 37.0	21.8 ± 26.2	0.263
SAM	10 (18.2)	6 (17.6)	0.592
HCM subtype			0.547
Septal	24 (43.6)	12 (35.3)	
Apical	7 (12.7)	8 (23.5)	
Concentric	3 (5.5)	2 (5.9)	
Septal/Apical	19 (34.5)	12 (35.3)	
Other	2 (3.6)	0	
CMR findings			
LVEDV (mL)	156.6 ± 185.7	186.1 ± 225.5	0.034
LVESV (mL)	41.4 ± 12.0	48.5 ± 22.1	0.052
LVEF (%)	68.4 ± 8.3	68.2 ± 7.6	0.891
Stroke volume (mL)	90.9 ± 23.7	99.4 ± 16.8	0.070
Cardiac output (L)	6.16 ± 1.64	6.58 ± 1.21	0.197
LV mass (g)	161.1 ± 48.4	183.4 ± 64.6	0.138
LV mass index (g/m^2^)	90.0 ± 23.5	100.1 ± 35.3	0.145
Presence of LGE	51 (92.7)	26 (76.5)	0.029 *
LGE volume (mL)	13.4 ± 12.5	11.0 ± 11.7	0.159
LGE volume percent (%)	9.12 ± 6.7	6.06 ± 4.9	0.021 *

Student’s t-test, Mann–Whitney test or χ2-test for detected vs. not detected (* *p*-value < 0.05). CMR: cardiac magnetic resonance, HCM: hypertrophic cardiomyopathy, IVSd: interventricular septum thickness at end diastole, LGE: late gadolinium enhancement, LAVI: left atrial volume index, LV: left ventricular, LVEDD: LV end-diastolic dimension, LVEDV: LV end-diastolic volume, LVEF: LV ejection fraction, LVESD: LV end-systolic dimension, LVESV LV end-systolic volume, LVOT: LV outflow tract, LVPWd: LV posterior wall at end diastole, MV: mid ventricular, RV: right ventricular, SAM: systolic anterior motion of mitral valve, SD: standard deviation.

**Table 5:** Major adverse cardiac and cerebrovascular events and interventions during follow-up.

Variables	Detected (*n* = 55)	Not detected (*n* = 34)	*p*-Value
	Mean ± SD or Number (%)		
MACCE	21 (38.2)	4 (11.8)	0.008 *
All cause death	0	0	
Sudden cardiac death	0	0	
Sustained VT/VF	4 (7.3)	0	
Worsening HF driven hospitalization	5 (9.1)	2 (5.9)	
New onset AF driven hospitalization	7 (12.7)	0	
Stroke	1 (1.8)	1 (2.9)	
Syncope	3 (5.5)	1 (2.9)	
AMI	1 (1.8)	0	
Interventions	9 (16.4)	1 (2.9)	0.082
ICD implantation	4 (7.2)	0	
Septal myectomy	6 (10.9)	1 (2.9)	
Heart transplantation	1 (1.8)	0	

Student’s t-test or χ2-test for detected vs. not detected (* *p*-value < 0.05). AF: atrial fibrillation, AMI: acute myocardial infarction, HF: heart failure, ICD: implantable cardioverter defibrillator.

## Supplementary Materials

The following are available online at https://www.mdpi.com/2077-0383/9/6/1671/s1, Table S1: Medication prescribed during follow-up.
