# Supplementary material for: Genotype-Related Clinical Characteristics and Myocardial Fibrosis and Their Association with Prognosis in Hypertrophic Cardiomyopathy
Source: J Clin Med. 2020 Jun 1;9(6):1671. doi: 10.3390/jcm9061671 (PMC7356272; doi:10.3390/jcm9061671)
Supplement: Supplementary file 1 [file jcm-09-01671-s001.pdf]

Supplementary material

Article

Genotype-Related Clinical Characteristics and Myocardial Fibrosis and their Association with Prognosis in Hypertrophic Cardiomyopathy

Hyung Yoon Kim [1,†], Jong Eun Park [2,†], Sang-Chol Lee [3,*], Eun-Seok Jeon [3], Young Keun On [3], Sung Mok Kim [4], Yeon Hyeon Choe [4], Chang-Seok Ki [5], Jong-Won Kim [6] and Kye Hun Kim [1]

[1] Department of Cardiovascular Medicine, Chonnam National University Medical School/Hospital, Gwangju, Korea; medoc7@gmail.com (H.Y.K.); christiankyehun@hanmail.net (K.H.K.)
[2] Department of Laboratory Medicine, Hanyang University Guri Hospital, Hanyang University College of Medicine, Guri, Korea; jongeun820@gmail.com
[3] Department of Internal medicine, Cardiovascular Imaging Center, Heart, Vascular & Stroke Institute; eunseok.jeon@samsung.com (E.-S.J.); yk.on@samsung.com (Y.K.O.)
[4] Department of Radiology, Cardiovascular Imaging Center, Heart, Vascular & Stroke Institute, Samsung Medical Center, Sungkyunkwan University School of Medicine, Seoul, Korea; sungmok_kim@hanmail.net (S.M.K.); ychoe11@gmail.com (Y.H.C.)
[5] Green Cross Genome, Yongin, Korea; changski.md@gmail.com
[6] Department of Laboratory Medicine and Genetics, Samsung Medical Center, Sungkyunkwan University School of Medicine, Seoul, Korea; kimjw@skku.edu
* Correspondence: chrislee.echo@gmail.com; Tel.: +82-2-3410-3419
† These authors equally contributed to this work as co-first authors.

Supplementary Table S1. Medication prescribed during follow-up

Variables	Detected (n=55)	Not detected (n=34)	*P*-value
	Mean ± SD or number (%)		
Amiodarone	0	0	NS
Beta-blocker	44 (80.0)	27 (79.4)	0.946
Calcium channel blocker	8 (14.5)	6 (17.6)	0.768
ACEi	1 (1.8)	0	0.618
ARB	8 (14.5)	8 (23.5)	0.395
Statin	3 (5.5)	4 (11.8)	0.421
Warfarin	2 (3.6)	1 (2.9)	0.676
Aspirin	21 (38.2)	17 (50.0)	0.378
